# The HPLC assay of concentration of azithromycin from two different manufacturers in gingival crevicular fluid (GCF)

**Published:** 2016

**Authors:** Mahmoud Khosravi-Samani, Khashayar Dehshiri, Sohrab Kazemi, Mohamadreza Shiran, Ali-Akbar Mohgadamnia

**Affiliations:** 1 Dental Materials Research Center, Department of Periodontology, Babol University of Medical Sciences, Babol, Iran.; 2 Neurosciences Research Center, Department of Pharmacology, Babol University of Medical Sciences, Babol, Iran.; 3 Department of Pharmacology, Mazandaran University of Medical Sciences, Sari, Iran.

**Keywords:** Azithromycin, Pharmacokinetics, HPLC, Gingival crevicular fluid.

## Abstract

**Background:**

Azithromycin (AZM) is used in periodontal infections. The present study compared gingival crevicular fluid concentration of azithromycin of two pharmaceutical companies through the HPLC method.

**Methods:**

Two groups (n=15) of healthy volunteers participated in this study. The first group received an imported azithromycin (ImAZM) tablet (250 mg, PO) and the second group received an azithromycin tablet (250 mg PO) manufactured by an Iranian pharmaceutical company (IrAZM). Intrasulcular paper points (#30) were used in inter-proximal areas of molars and canines to collect gingival crevicular fluid samples at 6, 12, 36, 84 and 156 hours after drug administration.

**Results:**

The maximum concentration of AZM in gingival crevicular fluid was detected in each group 36 hour after administration. The concentration levels for the participants receiving ImAZM and IrAZM were 14.38±5.75 and 12.64±3.53 ng/mL, respectively. The pharmacokinetic (PK) modeling data showed half-life of AZM was 107.47 hr & 91.42 hr while the clearance was 113.02 hr &119.0 hr for the group receiving ImAZM and IrAZM, respectively. No significant differences were observed in other PK parameters, areas under the concentration time curves for the groups were almost identical.

**Conclusion:**

According to the results, there were no significant differences between the PK parameters of ImAZM and IrAZM products. It may be concluded that different doses of AZM have relatively similar PK parameters among the healthy participants.

Azithromycin (AZM) is a macrolide antibiotic similar to erythromycin but has stronger effects, more persistent and more stable in acidic solutions with higher concentration in connective tissue. These properties have made azithromycin one of the best-selling antibiotics (1). This drug is commonly used in the treatment of many types of infections such as upper respiratory infection, otitis media middle ear infection, sexually transmitted infections, trachoma infection and treatment of refractory periodontal diseases associated with *P. gingivalis*. In addition, it is effective against pathogens responsible for periodontitis (2). AZM is also effective in gingival enlargement associated with cyclosporine (3). Another advantage of AZM is that frequent doses are not needed or necessarily used because of its long half-life (4).

The previous study had confirmed that long-term AZM therapy was more effective than other drug regimen in chronic adult periodontitis (5). There are two protocols to achieve therapeutic doses for periodontitis. The first one is the use of a loading dose of azithromycin, 500 mg on the first day and 250 mg for 2‒5 days; the second protocol includes the use of 500 mg of azithromycin for 3 days. Azithromycin is effective on anaerobic gram-negative bacilli. After the administration of 500 mg of azithromycin for 3 consecutive days, considerable levels of azithromycin are found in different tissues on day 7 to 10 (6, 7). 

AZM is rapidly dissolved in an acidic solution and converted to anhydroerythromycin, a degradation product of azithromycin which is very difficult to identify (8, 9). AZM is concentrated in neutrophils, macrophages, and especially in fibroblasts. All these cells play an active role in majority of periodontal infections (2). Significantly, higher concentration of AZM has been observed in samples of periodontal defects when compared to healthy tissue samples (10). AZM inhibits the synthesis of a specific protein that is essential for the survival of the bacteria. The maximum concentration of AZM in the tissues can be achieved on days 2 and 3 (11). 

It is effective against *A. actinomycetemcomitans*, *P. gingivalis* and other anaerobic gram-negative bacteria. AZM can reduce pocket depth and increase attachment gain, in response to the scaling in patients with periodontitis (2). After the administration of injectable azithromycin, this was detected for 2.3 to 4 hours in the plasma, with (70 hours) half-life. Plasma peak concentration is achieved 5 hours after oral intake, with a half-life of 59 hours. Taking azithromycin capsules with food decreases its absorption up to 52% and no particular difference is found in the effect of AZM between males and females (12). 

It is possible to evaluate AZM concentrations in the saliva, gingival crevicular fluid (GCF), and blood by some techniques, including *Micrococcus luteus* NCTC 8440 assay, HPLC with fluorescence (13, 14) mass spectrometry (9, 15) and UV (16, 17) detectors.

Evaluating the drug concentration in target tissues using sensitive and accurate methods such as HPLC and calculation of the PK parameters deems necessary due to the ever-increasing use of AZM in dental treatments. The present study was conducted in an attempt to compare the bioequivalence of domestic and imported azithromycin products and evaluating its concentration levels in GCF.

## Methods

***Subjects: ***This study was approved by the Ethics Committee of Babol University of Medical Sciences (Babol, Iran) and was recorded in IRCT (Iranian Registry of Clinical Trials) data bank with registration number: IRCT201412033451N2. Two groups of healthy volunteers participated in this study. The mean age of participants was in the range of 25±3 years. Twenty-one of the participants were males and nine were females. The mean age (±SD) was 24 (±2.31) and 24(±3.1). All participants were healthy volunteers with no specific diseases from the province of Mazandaran (northern Iran). Most of the participants were university students. The first group consisted of 11 males and 4 females aged 24.5±3.75 and 25.5±5.7 years, respectively. 

The participants’ total body weight was 76.66±7.2 kg; males and females weighing 75.63±7.44 and 71.75±7.6 kg, respectively. The mean total BMI of the subjects was 25.98±3.29, with 26.54±3.9 and 28.6±5.6 in males and females, respectively. 

The second group consisted of 10 males and 5 females aged 24±1.38; males and females aged 24.2±1.4 and 23.8±1.38, respectively. The subjects had a total average weight of 75.6±9.3, males and females weighing 76.4±8.4 and 74.2±11.8, with a total BMI of 24.5±3.34, with 24.3±2.74 and 25±4.6, respectively.

Exclusion criteria included taking any medication in previous two weeks, allergy to azithromycin, gingivitis, periodontitis, and evidence of gingival recession. A single dose of azithromycin (a 250-mg tablet) was administered using the imported azithromycin (ImAZM) and azithromycin produced by an Iranian manufacturer (IrAZM) in the first and second groups, respectively. Then the samples of GCF were collected at 6, 12, 36, 84 and 156 hours after administration of AZM and its concentrations were evaluated. Single dose of azithromycin in non-allergic subjects is safe; does not cause problems as it is used as prophylaxis in healthy individuals (18). 

After cleaning the patients' oral cavity using a strong suction, each sampling area was isolated by cotton rolls and dried using a gentle stream of air for 5 seconds. By doing so, it made possible to collect samples from the GCF in each step. 

A #30 paper point was placed slowly into the crevice of molar and canine teeth (1mm) to avoid mechanical injury (sulcular epithelium or blood contamination) and was left until 5-6 mm of its length was soaked with GCF. 

A fine straight forcep was used to remove the paper point slowly and placed it in tubes containing 0.5 mL of mobile phase solvent (methanol+buffer) (19,20). Each test tube was gently shaken several times. The samples were stored in the refrigerator at 4°C to prevent degradation of azithromycin prior transferring to a laboratory and measuring concentration levels applying HPLC technique (high performance liquid chromatography). The HPLC method used the following specifications: C18 column with 4.6 mm diameter and 25 mm length, UV detector wavelength of 210 nm.

## Results

**Figure 1:**
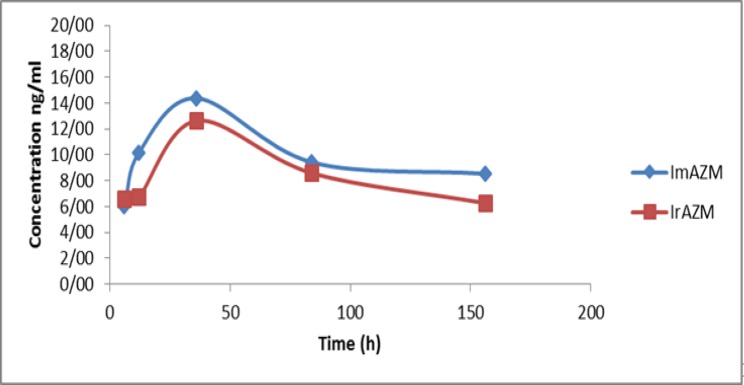
The figure displays the mean and standard deviation of concentrations (ng/mL) versus time (hours) in two groups. The concentration was measured by HPLC method and the number of subjects in each group was 15. Azithro_1: ImAZM, Azithro_2: IrAZM

***Azithromycin concentrations: ***Comparison of azithromycin concentration did not show a statistical difference between the two groups (figure 1) instead a higher concentration was observed in group IrAZM 6h after dosing. Azithromycin concentration in GCF increased rapidly to the maximum level after 36 hours (two days) but decreased at a higher rate up to 84 hours (day 4). The maximum and minimum concentrations of azithromycin at 6 h were 9.89 and 3.04 ng/ml for ImAZM and 16.490 and 2.67 ng/ml for IrAZM groups, respectively (table 1).

**Table 1:** Compartison of the PK parameters of two groups receiving ImAZM (imported azithromycin) and IrAZM(Iranian azithromycin)

**Treatment Groups**		**Time of Sampling (h)**					**Mean Tmax** ** (h)**	**Mean Cmax** **(ng/ml)**	**Mean AUC** **(0-156)**
		**6h**	**12h**	**36h**	**84h**	**156h**			
ImAZM	mean concentration (ng/ml)	6.017	10.160	14.388	9.427	8.545	36	14.388	1560.78
	SD	1.785	4.255	5.754	5.425	7.137			
	MIN	3.038	4.786	9.102	3.572	2.751			
	MAX	9.890	17.779	28.710	24.112	24.213			
	F (MAX/MIN)	3.26	3.71	3.15	6.75	8.80			
IrAZM	Mean concentration (ng/ml)	6.637	6.734	12.640	8.591	6.272	36	12.64	1317.03
	SD	4.002	4.208	3.533	4.507	3.572			
	MIN	2.668	3.107	6.427	4.218	2.050			
	MAX	16.490	17.114	19.356	20.717	13.888			
	F (MAX/MIN)	6.18	5.51	3.01	4.91	6.77			

Figure 1 shows that despite a higher concentration of azithromycin at 6 h in group IrAZM, this was reversed at 36 for ImAZM) receiving group. Fortunately, none of the participants in both groups complained of any specific symptoms during the study.

**Figure 2:**
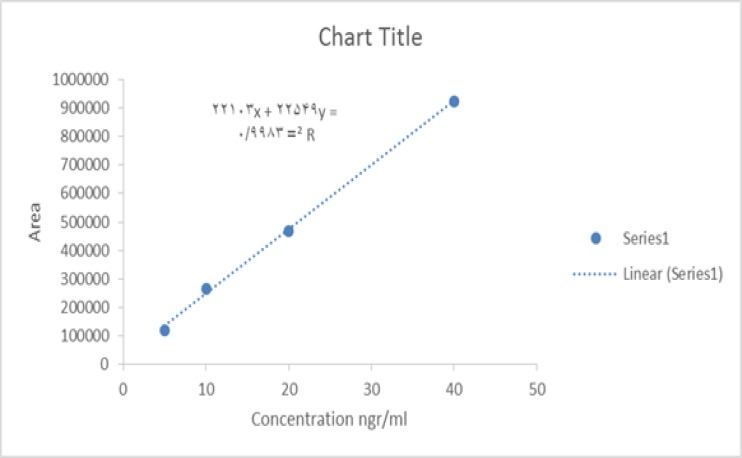
Calibration curve of peak area versus concentration of standard azithromycin solutions (ng/ml). Each point repeated at least three times. Acetaminophen (10 ng/ml).

***Standard curve: ***To determine the concentration of the GCF samples, a standard curve was plotted with at least 4 concentrations of azithromycin in standard solution (a gift from Chemidarou Pharmaceutical Company, Tehran, Iran) with acetaminophen concentration for all solutions as internal standard (figure 2). Standard peaks of azithromycin of 5, 10, 20 and 40 ng/mL and internal standard acetaminophen (10 ng/mL) were prepared. A standard curve was obtained using the peak area under the curve (chromatograms 1 and 2). 

R^2^ index (linearity) was also depicted in the chart. Coefficient of variation (CV) for the peaks was obtained based on area under the curve was 3.75%.

**Chromatogram 1:**
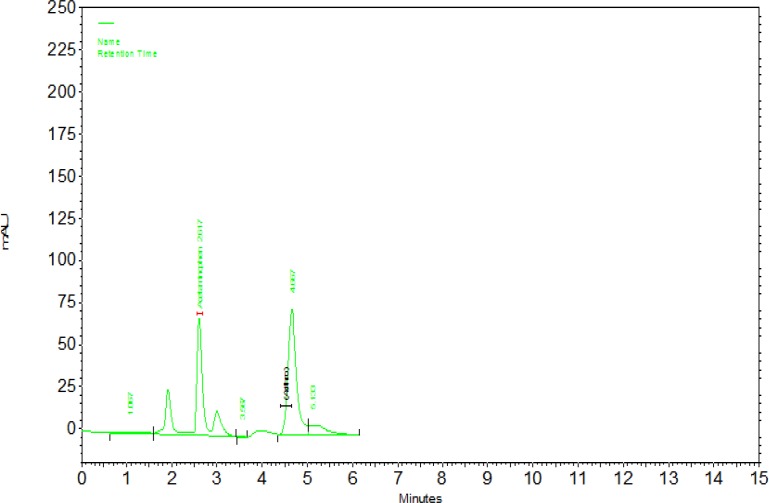
Chromatogram of the standard azithromycin solution at a concentration of 40 ng/mL (4.8min) in association with internal standard of acetaminophen at a concentration of 10 ng/ml (2.7min).

**Chromatogram 2:**
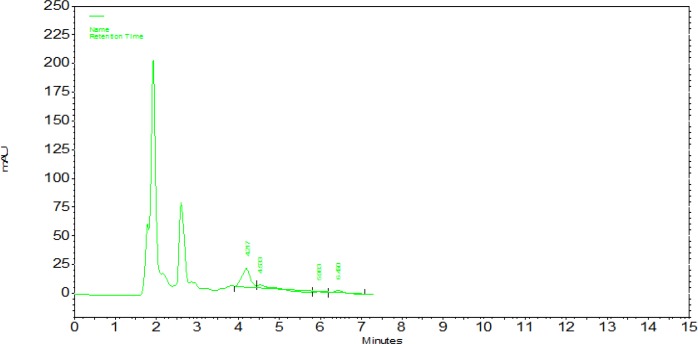
Azithromycin in serum chromatogram peak and internal acetaminophen concentration at 10 ng/ml.

***Data analysis with P-Pharm: ***A two-compartment PK model was assumed for analyzing concentration data obtained in GCF and for modeling P-Pharm, 1.5 software was used. The striping capability of the software allowed the users to record the subject’s data, extract initial hypothetical parameters and use as input for subsequent modeling.

**Figure 3:**
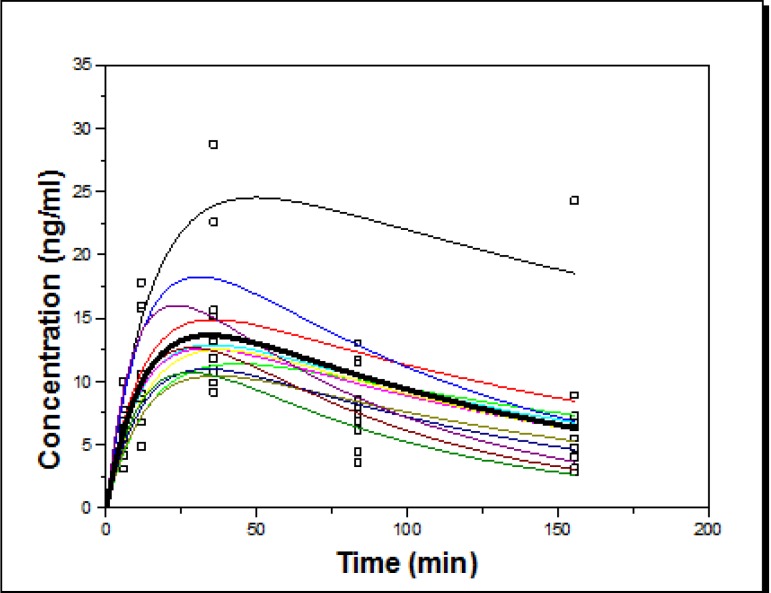
The relationship between azithromycin concentrations vs. time in each subject (individual fitting) in ImAZM group after PK modeling based on two-compartment oral kinetics

***The modeling of GCF concentration of azithromycin versus time: ***The results of the PK modeling based on the two-compartment kinetics following oral administration are shown as follows:

**A) Concentration versus time curve in ImAZM group:** In figure 3, the relationship between concentration versus time for each subject is shown in ImAZM group. According to figure 3, only one participant showed scattered levels at 84 and 156 h. 

**Figure 4:**
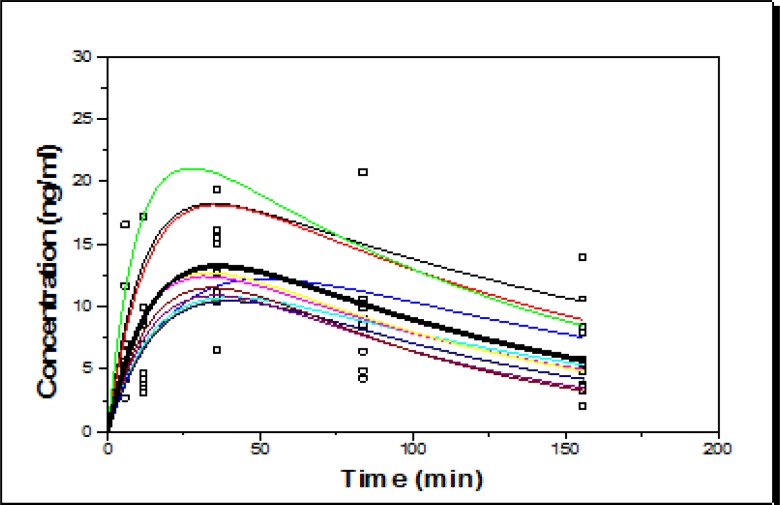
The relationship between azithromycin concentrations vs. time in each subject (individual fitting) in IrAZT group after PK modeling based on two-compartment oral kinetics

**B) Concentration versus time curve in IrAZM group:** In figure 4, the relationship between concentrations versus time for each subject is shown in IrAZM group.

**Figure 5:**
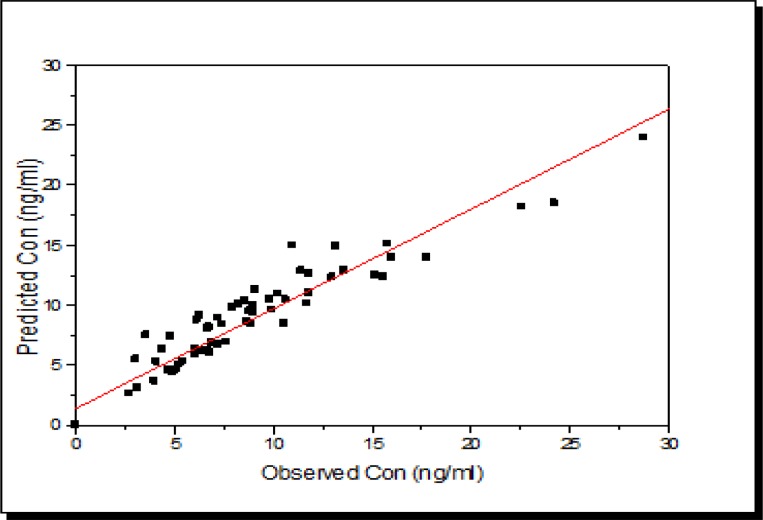
The relationship between population-based individual Bayesian predictions vs. observed plasma concentrations of azithromycin to the best-fit PK models applied to combined data from data of ImAZM group. The solid line represents a perfect match of the values

**Figure 6.**
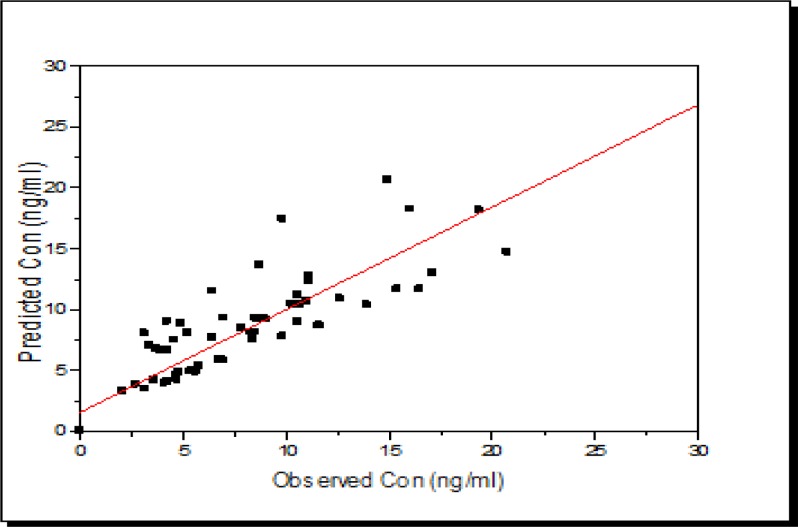
The relationship between population-based individual Bayesian predictions vs. observed plasma concentrations of azithromycin to the best-fit PK models applied to combined data from data of IrAZM group. The solid line represents a perfect match of the values

According to figure 4, scattered concentrations are seen at 84 and 156 h after drug administration. The peak concentration of azithromycin in GCF samples was recorded 12 h after taken (see figures 3 and 4). There was also an acceptable linear relationship between the measured and expected concentrations in both groups, as shown in figures 5 and 6. 

According to the figures, the model established an acceptable correlation between the measured and expected data.

To compare the data between the two groups in each variable, a non-paired t-test was used. Data analysis showed no statistically significant differences between any of the parameters. In addition, comparison of changes in concentration in each group in terms of sampling interval within and between groups using repeated-measures ANOVA showed significant differences in concentration only within each group over time (p<0.0001). 

Moreover, comparison of areas under the concentration curve (AUC0-156) versus time using the trapezoidal method with a t-test showed no significant differences between the two groups.

## Discussion

Periodontal disease is a serious disease in dentistry. Advanced stages lead to the loss of attachment, bone, and ultimately teeth loss. A major etiologic factor is pathogenic microorganisms. Azithromycin can be a good choice for the treatment of this disease (1). The GCF levels of azithromycin did not exhibit statistically significant differences between ImAZM and IrAZM groups; however, it appears that the results were better during the early hours of day 1 in ImAZM group, but later (days 2, 3, 5 and 7) better results were observed in IrAZM group, which might indicate a comparable quality product in IrAZM group.

The concentration of azithromycin at 36-hour interval (day 2) was the maximum amount, contrary to other studies, in which the highest concentration was recorded at 12 hours in plasma, gingiva, saliva and bone, which might be attributed to the selection of the lower dose of 250 mg compared to 500 mg during 3 days. However, the maximum observed dose was similar in the two studies (10). The concentration of azithromycin decreased on days 2 to 7, which is consistent with other studies, (4) that accumulation of macrolides in the gingival fibroblasts are large gingival. The results supported the hypothesis that the concentration of azithromycin will remain high in GCF for a long time. Based on the results, with the use of azithromycin, its concentration could be measured up to 7 days in GCF and it is comparable with other studies which was found in the serum and blood for 6.5 days.

These results could explain the hypothesis that cells lengthen the presence of oral azithromycin to boost its activity in the gingival tissue (4, 10). In another study, azithromycin was found in the serum 14 days after its systemic administration (4) Azithromycin has been shown to have a time-dependent effect. Antimicrobial effects of azithromycin are more dependent on duration of exposure rather than its concentrations in infected areas. This effect can be predicted by the duration of high concentration of azithromycin in tissues such as the GCF and gingiva (2). Azithromycin is used in two treatment protocols: a 5-day-administration of 250 mg in combination with loading dose or a 3-day administration of 500 mg of azithromycin (21). The latter is in relation with periodontal treatment, which may be due to the need for higher concentration in a shorter duration. Similarly, the results of the treatment are consistent with the highest concentration of azithromycin at 36 hours which was detected in this study.

Azithromycin concentrations range from 0.06 to 0.14 µg/mL, lower than the MIC (minimal inhibitory concentration) for *A. actinomycetemcomitans* (0.25-2.0 μg/mL), *P. gingivalis* (0.125-1 μg/mL) and *Prevotella intermedia* (0.03-1 ug/mL), indicating the need for higher concentration of azithromycin to combat microorganisms responsible for periodontal diseases (22, 23). Based on the results, when the PK parameters of two-compartment oral modeling were compared, no significant differences were found between the azithromycin tablets manufactured in Iran and those imported. In other words, from a pharmacokinetic point of view, there is bioequivalence between the two medicines with different manufacturers. 

Yet, despite apparent differences in their diffusion, there were no significant differences in clearance rates and half-lives between the two products, which may reflect the relatively high quality of manufacturing and the formulation of the domestic product. Given the high half-life and the concentrations of azithromycin observed in the GCF, it can be concluded that a single dose of azithromycin can result in optimal concentrations in the GCF. 

On the other hand, however, considering the low dose at which it is administered, this type of treatment may be appropriate for the maintenance phase or for the prevention of an infection spreading to surrounding tissues before gingival surgery.

**Conclusions: **According to the results, there were no significant differences in clearance rates and half-lives between ImAZM and IrAZM products. It may be concluded that different doses of AZM have relatively similar PK parameters among the healthy participants.

## Notes

**Funding:** This investigation has been financially supported by Research Affairs Division of Babol University of Medical Sciences (Grant no: 9337720).
